# Supplementary material for: Use of the International Classification of Diseases to Perinatal Mortality (ICD-PM) with verbal autopsy to determine the causes of stillbirths and neonatal deaths in rural Cambodia: a population-based, prospective, cohort study
Source: Lancet Reg Health West Pac. 2025 Jul 15;60:101626. doi: 10.1016/j.lanwpc.2025.101626 (PMC12282259; doi:10.1016/j.lanwpc.2025.101626)
Supplement: Appendices 1–8 [file mmc1.docx]

Appendix 1

Summary of verbal autopsy (VA) questionnaire adaptations.

Following the pilot we made the following adaptations to the WHO stillbirth and neonatal 2016 VA questionnaire to improve understanding and contextual acceptability:

- Reduced maximum recall period from the recommended one year, to six months as interviewers noticed that families were unable to recall events sufficiently after six months had elapsed.
- Question order made more chronological and logical.
- Repetitive questions removed.
- Translation of complex English phrasing improved.
- More space for open narrative at the beginning and end.
- Addition of questions about maternal and neonatal use of traditional medicine and practice.
- Removal of questions considered contextually inappropriate and a risk to establishing trust and rapport between interviewer and interviewee, for example:
  - Removal of questions directly asking about income, which the study team thought raised expectations about financial aid.
  - Removal of question about maternal use of illicit drugs in pregnancy, which the study team thought would breakdown trust and rapport between the interviewer and interviewee.
  - Removal of question about maternal marital status, which the study team thought could breakdown trust and rapport between the interviewer and interviewee. In Cambodia, particularly in rural areas, pregnancy outside of marriage is rare and stigmatised.

Appendix 2

ICD-PM codes, descriptions, definitions, and exemplar conditions, adapted and used to classify causes of stillbirths and neonatal deaths in our study.

| **Step 1** | **ICD-PM code** | | | **Description** | **Definition** | |
| --- | --- | --- | --- | --- | --- | --- |
|  | **U** | | | Unknown timing of death in relation to labour onset, died before delivery. | Unknown if signs of life at/after the onset of labour (fetal heart rate or fetal movement) and  no signs of life at delivery (no heart rate, no breathing, and no movement). | |
|  | **A** | | | Dead before/at the onset of labour, died before delivery. | No signs of life before/at the onset of labour (no fetal heart rate, no fetal movement) and  no signs of life at delivery (no heart rate, no breathing, and no movement). | |
|  | **I** | | | Alive at onset of labour, died before delivery. | Signs of life at/after the onset of labour (fetal heart rate and/or fetal movement) and  no signs of life at delivery (no heart rate, no breathing, and no movement). | |
|  | **N** | | | Alive at delivery, died before 28 days of life. | Any sign of life at/after delivery (any sign of heart rate, breathing, or movement). | |
|  |  |  |  |  |  |  |
| **Step 2** | **Timing of death** | | **ICD-PM code** | **Description** | **Example conditions** | **Further definition** |
|  | U | Stillbirth of unknown timing | **U1** | Congenital malformations, deformations, and chromosomal abnormalities | Major malformation eg. anencephaly, encephalocele, congenital hydrocephalus, spina bifida, etc. and/or presence of multiple malformations suggestive of syndrome eg. Trisomy 21. | Definite major malformation. |
|  |  |  | **U2** | In utero hypoxia | Hypoxic event / intrauterine hypoxia. |  |
|  |  |  | **U3** | Infection | Congenital syphilis, etc. |  |
|  |  |  | **U4** | Other specified in utero disorder |  |  |
|  |  |  | **U5** | Disorders related to fetal growth | Small for gestational age (<10th centile), large for gestational age (>90th centile), macrosomia (>4000g), post-term (>42 weeks gestation). |  |
|  |  |  | **U6** | In utero death of unspecified cause | No fetal condition identified. | Unknown – cannot definitively assign any other code. |
|  | A | Antepartum stillbirth | **A1** | Congenital malformations, deformations, and chromosomal abnormalities | Major malformation eg. anencephaly, encephalocele, congenital hydrocephalus, spina bifida, etc. and/or presence of multiple malformations suggestive of syndrome eg. Trisomy 21. | Definite major malformation. |
|  |  |  | **A2** | Infection | Congenital syphilis, etc. |  |
|  |  |  | **A3** | Antepartum hypoxia | Hypoxic event / intrauterine hypoxia. |  |
|  |  |  | **A4** | Other specified antepartum disorder | Cord rupture, twin-twin transfusion. |  |
|  |  |  | **A5** | Disorders related to fetal growth | Small for gestational age (<10th centile), large for gestational age (>90th centile), macrosomia (>4000g), post-term (>42 weeks gestation). |  |
|  |  |  | **A6** | Antepartum death of unspecified cause | No fetal condition identified. | Unknown – cannot definitively assign any other code. |
|  | I | Intrapartum stillbirth | **I1** | Congenital malformations, deformations, and chromosomal abnormalities | Major malformation eg. anencephaly, encephalocele, congenital hydrocephalus, spina bifida, etc. and/or presence of multiple malformations suggestive of syndrome eg. Trisomy 21. | Definite major malformation. |
|  |  |  | **I2** | Birth trauma | Severe injury due to birth injury eg. cerebral or intraventricular haemorrhage, or major bone/skull fracture. | Definite severe brain injury or fracture that is not a result of complications of intrapartum events. |
|  |  |  | **I3** | Acute intrapartum event | Hypoxic event / intrauterine hypoxia. |  |
|  |  |  | **I4** | Infection | Congenital syphilis, etc. |  |
|  |  |  | **I5** | Other specified intrapartum disorder | Cord rupture, twin-twin transfusion. |  |
|  |  |  | **I6** | Disorders related to fetal growth | Small for gestational age (<10th centile), large for gestational age (>90th centile), macrosomia (>4000g), post-term (>42 weeks gestation). |  |
|  |  |  | **I7** | Intrapartum death of unspecified cause | No fetal condition identified. | Unknown – cannot definitively assign any other code. |
|  | N | Neonatal death | **N1** | Congenital malformations, deformations, and chromosomal abnormalities | Major malformation eg. anencephaly, encephalocele, congenital hydrocephalus, spina bifida, cleft lip and/or palate, congenital heart disease, etc. and/or presence of multiple malformations suggestive of syndrome eg. Trisomy 21. | Definite major malformation. |
|  |  |  | **N2** | Disorders related to fetal growth | Small for gestational age (<10th centile), large for gestational age (>90th centile), macrosomia (>4000g), post-term (>42 weeks gestation). | Definite growth restriction based on definite gestation and birth weight. |
|  |  |  | **N3** | Birth trauma | Severe injury due to birth injury eg. cerebral or intraventricular haemorrhage, or major bone/skull fracture. | Definite severe brain injury or fracture that is not a result of complications of intrapartum events. |
|  |  |  | **N4** | Complications of intrapartum events | Hypoxic event / intrauterine hypoxia, birth asphyxia **. | Include in this code any severe brain injury that is a result of complications of intrapartum events, including hypoxic ischaemic encephalopathy * |
|  |  |  | **N5** | Convulsions and disorders of cerebral status | Hypoxic ischaemic encephalopathy. | To be coded as N4 * |
|  |  |  | **N6** | Infection | Sepsis, congenital pneumonia, meningitis, tetanus neonatorum, congenital syphilis, omphalitis, skin infection, etc. | Term + danger sign.  Well and then deteriorate with at least 1 sign or symptom suggesting sepsis. |
|  |  |  | **N7** | Respiratory and cardiovascular disorders | Respiratory distress syndrome, meconium aspiration syndrome, pneumothorax, pulmonary haemorrhage, cardiac dysrhythmia, cardiac failure, etc. |  |
|  |  |  | **N8** | Other neonatal conditions | Necrotising enterocolitis, vomiting/feeding problem, kernicterus, haemolytic disease, hydrops fetalis, bleeding disorder, fetal blood loss, neonatal abstinence syndrome. | Include in this code any specified diagnoses defined in WHO N10 (Miscellaneous) and N11 (neonatal death of unspecified cause) codes. |
|  |  |  | **N9** | Low birth weight and prematurity | Low birth weight (<2500g) and / or prematurity (<37 weeks gestation) without definite more specific cause eg. respiratory distress syndrome, apneoa of prematurity, sepsis, necrotising enterocolitis, small for gestational age, etc. |  |
|  |  |  | **N10** | Miscellaneous |  | To be coded as N8 |
|  |  |  | **N11** | Neonatal death of unspecified cause | No neonatal condition identified. | Unknown – cannot definitively assign any other code.  Note: If any of the diagnoses from the WHO definition (congenital renal failure, neonatal abstinence syndrome, etc) – to be coded as N8 (other neonatal conditions) |
|  |  |  |  |  |  |  |
| **Step 3** |  |  | **ICD-PM code** | **Description** | **Example conditions** |  |
|  | Maternal contributing condition | | **M1** | Complications of placenta, cord and membranes | Placenta praevia, antepartum haemorrhage, cord prolapse/compression, chorioamnionitis. |  |
|  |  |  | **M2** | Maternal complications of pregnancy | Multiple pregnancy, maternal death, premature (<37 weeks gestation) rupture of membranes, etc. |  |
|  |  |  | **M3** | Other complications of labour and delivery | Premature (<37 weeks gestation) labour and delivery, Obstructed delivery due to breech/malpresentation/shoulder dystocia and/or obstructed delivery requiring forceps/vacuum/emergency caesarean. | Preterm labour and delivery (<37 weeks gestation) with no apparent underlying pathology (idiopathic). |
|  |  |  | **M4** | Maternal medical and surgical conditions | Maternal infection, maternal injury, maternal hypertension, preeclampsia or eclampsia, gestational diabetes mellitus, maternal use of tobacco, alcohol or drugs, etc. |  |
|  |  |  | **M5** | No maternal condition | No maternal condition identified. | Unknown – cannot definitively assign any other code. |
| * Differentiation between birth asphyxia (N4) and hypoxic ischaemic encephalopathy (HIE) (N5) was not possible. We felt N4 is the more useful code from a public health perspective as birth asphyxia occurs before HIE (the earlier event in the chain of events), is the broader term that includes HIE, and diagnosis involves identifying the cause of oxygen deprivation (compared to HIE which involved assessing severity of brain injury).  ** Since cardiac fetal monitoring was not available during labour, birth asphyxia was suspected based on the overall clinical picture (e.g. prolonged or obstructed delivery, impaired transition to extra-uterine life). | | | | | | |

Appendix 3

Relative risks of death (stillbirths and neonatal deaths) by characteristics of deliveries ≥28 weeks gestation.

|  | **Univariable** | | | | **Multivariable,** N = 23,100 | | |
| --- | --- | --- | --- | --- | --- | --- | --- |
| **Characteristic** | **N** | **RR**^1^ | **95% CI**^1^ | **p-value** | **RR**^1^ | **95% CI**^1^ | **p-value** |
| **Male sex** | 23,969 | 1.46 | 1.21, 1.76 | <0.001 | 1.45 | 1.19, 1.76 | <0.001 |
| **Preterm*** | 23,937 | 25.1 | 21.1, 29.8 | <0.001 | 9.65 | 7.14, 13.0 | <0.001 |
| **Low birth weight**** | 23,126 | 14.1 | 11.5, 17.2 | <0.001 | 3.37 | 2.48, 4.56 | <0.001 |
| **Multiple birth** | 23,972 | 4.97 | 3.36, 7.00 | <0.001 | 1.32 | 0.95, 1.82 | 0.10 |
| **Non-facility birth***** | 23,967 | 3.36 | 2.63, 4.25 | <0.001 | 2.32 | 1.80, 2.99 | <0.001 |
| ^1^RR = Relative Risk, CI = Confidence Interval  *Preterm defined as <37 weeks gestational age  **Low birth weight defined as <2500g  ***Home births and births enroute (on the way to a facility) | | | | | | | |

Appendix 4

Comparison of characteristics of deaths that were found by the surveillance system compared to the deaths that were found at the facility. Non-governmental organisation (NGO).

| **Characteristic** | **Surveillance**  N = 457 | **Facility records**  N = 65 | **Overall**  N = 522 | **p-value**^1^ |
| --- | --- | --- | --- | --- |
| **Timing of death, n (%)** |  |  |  | 0.042 |
| Stillbirth | 198/457 (43.3%) | 31/65 (47.7%) | 229/522 (43.9%) |  |
| Early neonatal death | 218/457 (47.7%) | 34/65 (52.3%) | 252/522 (48.3%) |  |
| Late neonatal death | 41/457 (9.0%) | 0/65 (0.0%) | 41/522 (7.9%) |  |
| **Male sex, n (%)** | 278/454 (61.2%) | 12/29 (41.4%) | 290/483 (60.0%) | 0.034 |
| Missing | 3 | 36 | 39 |  |
| **Preterm*, n (%)** | 232/456 (50.9%) | 19/42 (45.2%) | 251/498 (50.4%) | 0.484 |
| Missing | 1 | 23 | 24 |  |
| **Birth weight (kg), median (IQR)** | 2.30 (1.50, 3.00) | 1.95 (1.20, 2.90) | 2.30 (1.50, 3.00) | 0.028 |
| Missing | 88 | 25 | 113 |  |
| **Multiple birth, n (%)** | 28/457 (6.1%) | 3/65 (4.6%) | 31/522 (5.9%) | 0.785 |
| **Birth location, n (%)** |  |  |  | <0.001 |
| Home | 54/457 (11.8%) | 0/61 (0.0%) | 54/518 (10.4%) |  |
| Enroute | 22/457 (4.8%) | 3/61 (4.9%) | 25/518 (4.8%) |  |
| Primary | 170/457 (37.2%) | 19/61 (31.1%) | 189/518 (36.5%) |  |
| Secondary | 150/457 (32.8%) | 38/61 (62.3%) | 188/518 (36.3%) |  |
| Private | 6/457 (1.3%) | 0/61 (0.0%) | 6/518 (1.2%) |  |
| Other | 2/457 (0.4%) | 1/61 (1.6%) | 3/518 (0.6%) |  |
| Other province Primary | 6/457 (1.3%) | 0/61 (0.0%) | 6/518 (1.2%) |  |
| Other province Secondary | 7/457 (1.5%) | 0/61 (0.0%) | 7/518 (1.4%) |  |
| Other province Tertiary (NGO) | 38/457 (8.3%) | 0/61 (0.0%) | 38/518 (7.3%) |  |
| Other province Private | 2/457 (0.4%) | 0/61 (0.0%) | 2/518 (0.4%) |  |
| Missing | 0 | 4 | 4 |  |
| **Death location, n (%)** |  |  |  | <0.001 |
| Home | 77/259 (29.7%) | 0/34 (0.0%) | 77/293 (26.3%) |  |
| Enroute | 13/259 (5.0%) | 0/34 (0.0%) | 13/293 (4.4%) |  |
| Primary | 48/259 (18.5%) | 10/34 (29.4%) | 58/293 (19.8%) |  |
| Secondary | 74/259 (28.6%) | 24/34 (70.6%) | 98/293 (33.4%) |  |
| Private | 2/259 (0.8%) | 0/34 (0.0%) | 2/293 (0.7%) |  |
| Other | 2/259 (0.8%) | 0/34 (0.0%) | 2/293 (0.7%) |  |
| Other province Primary | 1/259 (0.4%) | 0/34 (0.0%) | 1/293 (0.3%) |  |
| Other province Tertiary (NGO) | 42/259 (16.2%) | 0/34 (0.0%) | 42/293 (14.3%) |  |
| Missing | 198 | 31 | 229 |  |
| ^1^Pearson's Chi-squared test; Wilcoxon rank sum test; Fisher's exact test  *Preterm defined as <37 weeks gestational age | | | | |

Appendix 5

Death cohort characteristics, stratified by timing of death.

|  | **Stillbirth (unknown timing)**  N = 75 | **Antepartum stillbirth**  N = 94 | **Intrapartum stillbirth**  N = 60 | **Early neonatal death**  N = 252 | **Late neonatal death**  N = 41 | **Overall**  N = 522 |
| --- | --- | --- | --- | --- | --- | --- |
| **Male sex,**  **n (%)** | 27/57 (47.4%) | 49/93 (52.7%) | 44/60 (73.3%) | 145/232 (62.5%) | 25/41 (61.0%) | 290/483 (60.0%) |
| Missing | 18 | 1 | 0 | 20 | 0 | 39 |
| **Preterm*,**  **n (%)** | 25/64 (39.1%) | 53/94 (56.4%) | 10/60 (16.7%) | 146/240 (60.8%) | 17/40 (42.5%) | 251/498 (50.4%) |
| Missing | 11 | 0 | 0 | 12 | 1 | 24 |
| **Low birth weight**,**  **n (%)** | 15/40 (37.5%) | 31/65 (47.7%) | 6/45  (13.3%) | 146/221 (66.1%) | 19/38 (50.0%) | 217/409 (53.1%) |
| Missing | 35 | 29 | 15 | 31 | 3 | 113 |
| **Non-facility birth***, n (%)** | 10/73 (13.7%) | 14/94 (14.9%) | 11/60 (18.3%) | 34/250  (13.6%) | 10/41 (24.4%) | 79/518 (15.3%) |
| Missing | 2 | 0 | 0 | 2 | 0 | 4 |
| *Preterm defined as <37 weeks gestational age  **Low birth weight defined as <2500g  ***Home births and births enroute (on the way to a facility) | | | | | | |

Appendix 6

Comparison of characteristics of verbal autopsy (VA) cases that were enrolled versus VA cases that were not enrolled. Non-governmental organisation (NGO).

| **Characteristic** | **Enrolled**  N = 413 | **Not enrolled**  N = 109 | **Overall**  N = 522 | **p-value**^1^ |
| --- | --- | --- | --- | --- |
| **Timing of death, n (%)** |  |  |  | 0.318 |
| Stillbirth | 175/413 (42.4%) | 54/109 (49.5%) | 229/522 (43.9%) |  |
| Early neonatal death | 203/413 (49.2%) | 49/109 (45.0%) | 252/522 (48.3%) |  |
| Late neonatal death | 35/413 (8.5%) | 6/109 (5.5%) | 41/522 (7.9%) |  |
| **Male sex, n (%)** | 255/412 (61.9%) | 35/71 (49.3%) | 290/483 (60.0%) | 0.045 |
| Missing | 1 | 38 | 39 |  |
| **Preterm*, n (%)** | 207/412 (50.2%) | 44/86 (51.2%) | 251/498 (50.4%) | 0.877 |
| Missing | 1 | 23 | 24 |  |
| **Birth weight (kg), median (IQR)** | 2.35 (1.55, 3.05) | 2.00 (1.30, 2.90) | 2.30 (1.50, 3.00) | 0.060 |
| Missing | 73 | 40 | 113 |  |
| **Multiple birth, n (%)** | 26/413 (6.3%) | 5/109 (4.6%) | 31/522 (5.9%) | 0.502 |
| **Birth location, n (%)** |  |  |  | <0.001 |
| Home | 43/413 (10.4%) | 11/105 (10.5%) | 54/518 (10.4%) |  |
| Enroute | 25/413 (6.1%) | 0/105 (0.0%) | 25/518 (4.8%) |  |
| Primary | 158/413 (38.3%) | 31/105 (29.5%) | 189/518 (36.5%) |  |
| Secondary | 143/413 (34.6%) | 45/105 (42.9%) | 188/518 (36.3%) |  |
| Private | 2/413 (0.5%) | 4/105 (3.8%) | 6/518 (1.2%) |  |
| Other | 0/413 (0.0%) | 3/105 (2.9%) | 3/518 (0.6%) |  |
| Other province Primary | 3/413 (0.7%) | 3/105 (2.9%) | 6/518 (1.2%) |  |
| Other province Secondary | 6/413 (1.5%) | 1/105 (1.0%) | 7/518 (1.4%) |  |
| Other province Tertiary (NGO) | 31/413 (7.5%) | 7/105 (6.7%) | 38/518 (7.3%) |  |
| Other province Private | 2/413 (0.5%) | 0/105 (0.0%) | 2/518 (0.4%) |  |
| Missing | 0 | 4 | 4 |  |
| **Death location, n (%)** |  |  |  | <0.001 |
| Home | 64/238 (26.9%) | 13/55 (23.6%) | 77/293 (26.3%) |  |
| Enroute | 13/238 (5.5%) | 0/55 (0.0%) | 13/293 (4.4%) |  |
| Primary | 48/238 (20.2%) | 10/55 (18.2%) | 58/293 (19.8%) |  |
| Secondary | 71/238 (29.8%) | 27/55 (49.1%) | 98/293 (33.4%) |  |
| Private | 0/238 (0.0%) | 2/55 (3.6%) | 2/293 (0.7%) |  |
| Other | 0/238 (0.0%) | 2/55 (3.6%) | 2/293 (0.7%) |  |
| Other province Primary | 0/238 (0.0%) | 1/55 (1.8%) | 1/293 (0.3%) |  |
| Other province Tertiary (NGO) | 42/238 (17.6%) | 0/55 (0.0%) | 42/293 (14.3%) |  |
| Missing | 175 | 54 | 229 |  |
| ^1^Pearson's Chi-squared test; Wilcoxon rank sum test; Fisher’s Exact Test.  *Preterm defined as <37 weeks gestational age | | | | |

Appendix 7

ICD-PM classification of all intrapartum stillbirths and early neonatal deaths combined.

|  |  | | **Maternal contributing condition** | | | | | **Total** |
| --- | --- | --- | --- | --- | --- | --- | --- | --- |
|  | **M1** | **M2** | | **M3** | **M4** | **M5** | **M** |  |
|  | **Complications of placenta, cord, membranes** | **Maternal complications of pregnancy** | | **Other complications of labour and delivery** | **Maternal medical and surgical conditions** | **No maternal condition identified** | **Unclassifiable** |  |
| **Hypoxic event** | 14 (5.3%) | 7 (2.7%) | | 69 (26.2%) | 1 (0.4%) | 13 (4.9%) | 4 (1.5%) | 108 (41.1%) |
| **Low birth weight and prematurity** | 9 (3.4%) | 4 (1.5%) | | 59 (22.4%) | 4 (1.5%) | 3 (1.1%) | 4 (1.5%) | 83 (31.6%) |
| **Infection** | 0 (0.0%) | 0 (0.0%) | | 3 (1.1%) | 4 (1.5%) | 24 (9.1%) | 3 (1.1%) | 34 (12.9%) |
| **Congenital malformations** | 0 (0.0%) | 1 (0.4%) | | 0 (0.0%) | 1 (0.4%) | 11 (4.2%) | 1 (0.4%) | 14 (5.3%) |
| **Other condition** | 0 (0.0%) | 0 (0.0%) | | 0 (0.0%) | 0 (0.0%) | 1 (0.4%) | 0 (0.0%) | 1 (0.4%) |
| **Unspecified cause** | 0 (0.0%) | 0 (0.0%) | | 1 (0.4%) | 1 (0.4%) | 12 (4.6%) | 0 (0.0%) | 14 (5.3%) |
| **Unclassifiable** | 0 (0.0%) | 0 (0.0%) | | 1 (0.4%) | 2 (0.8%) | 5 (1.9%) | 1 (0.4%) | 9 (3.4%) |
| **Total** | 23 (8.7%) | 12 (4.6%) | | 133 (50.6%) | 13 (4.9%) | 69 (26.2%) | 13 (4.9%) | 263 (100.0%) |

Appendix 8

Cumulative cause-specific mortality of neonatal deaths by age of death (n=238).

**
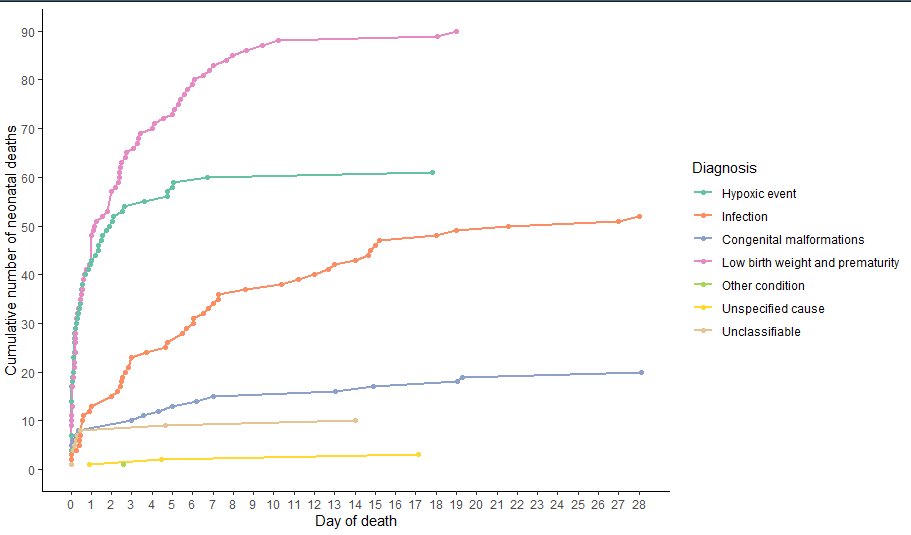
**
