# Supplementary material for: Use of the International Classification of Diseases to Perinatal Mortality (ICD-PM) with verbal autopsy to determine the causes of stillbirths and neonatal deaths in rural Cambodia: a population-based, prospective, cohort study
Source: Lancet Reg Health West Pac. 2025 Jul 15;60:101626. doi: 10.1016/j.lanwpc.2025.101626 (PMC12282259; doi:10.1016/j.lanwpc.2025.101626)
Supplement: Translated Abstract_Khmer [file mmc2.docx]

This translation in Khmer was submitted by the authors and we reproduce it as supplied. It has not been peer reviewed. Our editorial processes have only been applied to the original abstract in English, which should serve as reference for this manuscript.

ការប្រើប្រាស់ចំណាត់ថ្នាក់អន្តរជាតិនៃជំងឺអំពីការស្លាប់ជុំវិញកំណើត (ICD-PM) ជាមួយការធ្វើកោសល្យវិច័យដោយពាក្យសំដី​(VA)ដើម្បីកំណត់ពីមូលហេតុនៃការស្លាប់កើត និង​ កើតស្លាប់របស់ទារកនៅតំបន់ដាច់ស្រយ៉ាលនៃប្រទេសកម្ពុជា៖​ ការសិក្សាលើក្រុមប្រជាជនតាម (a population-based, prospective, cohort study)។

**សេចក្តីសង្ខេបនៃការសិក្សា**

សាវតា

ការស្លាប់ជុំវិញកំណើតនៅតែជាបញ្ហាសុខភាពប្រឈមដ៏សំខាន់ នៅជុំវិញពិភពលោក ជាពិសេសនៅប្រទេសដែលមានចំណូលកម្រិតទាប និងមធ្យម។ ទិន្នន័យអំពីមូលហេតុនៃការស្លាប់ដែលមានភាពត្រឹមត្រូវគឺមានសារៈសំខាន់ណាស់សម្រាប់ការចាត់ចែងអន្តរាគមន៍ដែលមានប្រសិទ្ធភាព ប៉ុន្តែជាញឹកញាប់វាមានភាពខ្វះខាត។ ការសិក្សានេះមានគោលបំណងកំណត់មូលហេតុនៃការស្លាប់កើត និង កើតស្លាប់របស់ទារកនៅតំបន់ដាច់ស្រយ៉ាលនៃប្រទេសកម្ពុជាដោយប្រើប្រាស់ ការធ្វើកោសល្យវិច័យដោយពាក្យសំដី និង ប្រើប្រាស់ចំណាត់ថ្នាក់អន្តរជាតិនៃជំងឺអំពីការស្លាប់ជុំវិញកំណើតនៃអង្គការសុខភាពពិភពលោក (ICD-PM)។

វិធីសាស្រ្ត

ការសិក្សាជាមុន​(Prospective study) រយៈពេល៤ឆ្នាំ (2018-2022) នៅខេត្តព្រះវិហារ​ ប្រទេសកម្ពុជា បានបង្កើតឡើងនូវប្រព័ន្ធតាមដានស្រ្តីមានផ្ទៃពោះ ដោយក្រុមទ្រទ្រង់សុខភាពភូមិ។ ការធ្វើកោសល្យវិច័យដោយពាក្យសំដីត្រូវបានអនុវត្តលើទារកស្លាប់កើត និង កើតស្លាប់ ដោយមានការវិភាគដោយពីវេជ្ជបណ្ឌិតពីររូបដើម្បីធ្វើការបកស្រាយនូវទិន្នន័យកោសល្យវិច័យដោយពាក្យសំដី។​​ ចំណាត់ថ្នាក់អន្តរជាតិនៃជំងឺអំពីការស្លាប់ជុំវិញកំណើត (ICD-PM) ត្រូវបានប្រើសម្រាប់ចាត់ថ្នាក់មូលហេតុនៃការស្លាប់ ដោយមានការសម្របសម្រួលផ្លាស់ប្ដូរផ្នែកខ្លះសម្រាប់ករណីទារកស្លាប់ដែលមិនអាចកំណត់ពេលវេលានៃការស្លាប់បាន។

លទ្ធផល

ករណីស្លាប់សរុប 522 នាក់ (229​​ ករណីស្លាប់កើត​ និង293ករណីកើតស្លាប់) ត្រូវបានកត់ត្រា និង 79,1%​(413ករណី) ត្រូវបានធ្វើកោសល្យវិច័យដោយពាក្យសំដី។ ដោយយោងទៅតាម ICD-PM បានកំណត់មូលហេតុជាបឋមនៃការស្លាប់របស់ទារកចំនួន​36,6%ជាទារកស្លាប់កើត និង 95% ជាទារកកើតស្លាប់។​ មូលហេតុចំបងនៃការស្លាប់គឺ ទារកស្លាប់កើតដោយសារកង្វះអុកស្សីហ្សែនកំឡុងពេលឈឺពោះសម្រាល (78,3%) ទារកមានទម្ងន់ទាប និងទារកកើតមិនគ្រប់ខែចំពោះការស្លាប់របស់ទារកកំឡុងអាយុមួយសប្តាហ៍ដំបូងនៃជីវិត (40,9%) និង ការឆ្លងរោគចំពោះទារកមានអាយុលើសពីមួយសប្តាហ៍​(51,4%)។ ផលវិបាកកំឡុងពេលឈឺពោះសម្រាល និង ពេលសម្រាលគឺជាកត្តាចំបងដែលរួមចំណែកមកពីស្ថានភាពរបស់ម្តាយដែលធ្វើអោយមានទារកស្លាប់កើតកំឡុងពេលឈឺពោះសម្រាល (63,3%) និង ទារកស្លាប់ក្នុងកំឡុងអាយុ១សប្តាហ៍ដំបូង (42,4%)។ ចំពោះទារកស្លាប់កើតដែលមិនអាចបញ្ជាក់ពីពេលវេលាមានចំនួន12%។​

ការបកស្រាយ

ការប្រើប្រាស់ប្រព័ន្ធ ICD-PM ជាមួយនឹងទិន្នន័យដែលបានស្រង់ពីកោសល្យវិច័យដោយពាក្យសំដី​ (VA) បានផ្តល់នូវការយល់ដឹងដ៏មានតម្លៃទៅលើមូលហេតុនៃទារកស្លាប់កើត និង ទារកកើតស្លាប់។ ទោះបីជាយ៉ាងណាការសម្របសម្រួលមួយចំនួនគឺចាំបាច់ក្នុងការដោះស្រាយអំពីភាពនៅមានកម្រិតរបស់ប្រព័ន្ធ ICD-PM ជាពិសេសក្នុងការចាត់ចំណាត់ថ្នាក់ករណីដែលមិនអាចកំណត់ពេលវេលានៃការស្លាប់របស់ទារកបាន។ លទ្ធផលនៃការសិក្សារបស់យើងអាចរួមចំណែកក្នុងកិច្ចខិតខំប្រឹងប្រែងជាសកលក្នុងការធ្វើឱ្យប្រសើរឡើងនូវការរាយការណ៍ទិន្នន័យការស្លាប់ជុំវិញកំណើត។
